# *Drd4* gene polymorphisms are associated with personality variation in a passerine bird

**DOI:** 10.1098/rspb.2007.0337

**Published:** 2007-05-01

**Authors:** Andrew E Fidler, Kees van Oers, Piet J Drent, Sylvia Kuhn, Jakob C Mueller, Bart Kempenaers

**Affiliations:** 1 Max-Planck Institute for OrnithologyPO Box 1564, 82319 Starnberg, Germany; 2 Cawthron InstitutePrivate Bag 2, Nelson 7042, New Zealand; 3 Netherlands Institute of EcologyPO Box 40, 6666 ZG Heteren, The Netherlands

**Keywords:** personality, *Drd4*, dopamine receptor, polymorphisms, *Parus major*, novelty seeking

## Abstract

Polymorphisms in several neurotransmitter-associated genes have been associated with variation in human personality traits. Among the more promising of such associations is that between the human dopamine receptor D4 gene (*Drd4*) variants and novelty-seeking behaviour. However, genetic epistasis, genotype–environment interactions and confounding environmental factors all act to obscure genotype–personality relationships. Such problems can be addressed by measuring personality under standardized conditions and by selection experiments, with both approaches only feasible with non-human animals. Looking for similar *Drd4* genotype–personality associations in a free-living bird, the great tit (*Parus major*), we detected 73 polymorphisms (66 SNPs, 7 indels) in the *P. major Drd4* orthologue. Two of the *P. major Drd4* gene polymorphisms were investigated for evidence of association with novelty-seeking behaviour: a coding region synonymous single nucleotide polymorphism (SNP830) and a 15 bp indel (ID15) located 5′ to the putative transcription initiation site. Frequencies of the three *Drd4* SNP830 genotypes, but not the ID15 genotypes, differed significantly between two *P. major* lines selected over four generations for divergent levels of ‘early exploratory behaviour’ (EEB). Strong corroborating evidence for the significance of this finding comes from the analysis of free-living, unselected birds where we found a significant association between SNP830 genotypes and differing mean EEB levels. These findings suggest that an association between *Drd4* gene polymorphisms and animal personality variation predates the divergence of the avian and mammalian lineages. Furthermore, this work heralds the possibility of following microevolutionary changes in frequencies of behaviourally relevant *Drd4* polymorphisms within populations where natural selection acts differentially on different personality types.

## 1. Introduction

In recent years, animal personalities, or ‘behavioural syndromes’, have become the subject of increasing scientific investigation (Gosling 2001; Dall *et al*. 2004; Sih *et al*. 2004; Groothuis & Carere 2005; Bell 2007). Aside from being of intrinsic interest, personality can influence how individual animals cope with both predictable and stochastic environmental variation and, consequently, how animal populations may adaptively evolve (Dingemanse *et al*. 2004; Both *et al*. 2005; Dingemanse & Réale 2005). To better understand the ecological and evolutionary significance of personality variation in natural, free-living animal populations, a greater understanding of the underlying molecular genetic mechanisms is needed.

Studies reported over the past decade indicate that polymorphisms in the *Drd4* gene may be associated with variation in measures of novelty-seeking behaviour in humans (reviewed in Kluger *et al*. 2002; Schinka *et al*. 2002; Reif & Lesch 2003; van Gestel & Van Broeckhoven 2003; Savitz & Ramesar 2004; Ebstein 2006). However, culture and environment play such a large role in determining individual human behaviours that it is to be expected, that genetic contributions to interindividual behavioural differences will be relatively modest. The importance of learned behaviour, along with the complexities of human population genetic structuring, has been evoked to account for inconsistencies and contradictions in the many replicate studies of associations between *Drd4* gene polymorphisms and human personality parameters (Kluger *et al*. 2002; Schinka *et al*. 2002; Reif & Lesch 2003; van Gestel & Van Broeckhoven 2003; Savitz & Ramesar 2004; Ebstein 2006).

Therefore, non-human vertebrates may prove useful subjects for genotype–personality phenotype association studies as, in these species, ‘cultural’ determinants of behaviour are not expected to be as strong as among humans. Different breeds of domesticated animals (chickens (*Gallus gallus*), Sugiyama *et al*. 2004; dogs (*Canis familiaris*), Ito *et al*. 2004) have been found to have differing frequencies of *Drd4* alleles, while studies of both domesticated horses (*Equus caballus*) and captive monkeys (*Cercopithecus aethiops*) have reported associations between personality parameters and *Drd4* gene polymorphisms (Momozawa *et al*. 2005; Bailey *et al*. 2007). Furthermore, *Drd4* gene polymorphisms have been reported for free-living species (Mogensen *et al*. 2006), but studies associating this variation with behaviour are absent.

Notwithstanding the publication of a draft jungle fowl (*Gallus gallus*) genomic sequence (International Chicken Genome Sequencing Consortium 2004), avian genomic studies are in their infancy when compared with those of mammals, particularly humans. Our present knowledge of polymorphic variation in potentially behaviour-related genes of birds, other than the domestic chicken (Keeling *et al*. 2004; Natt *et al*. 2007), is very limited. However, the well-established advantages of birds as subjects for ethological and behavioural ecological research indicate that these avian genomic challenges are worth confronting.

The great tit (*Parus major*) is emerging as a significant model species for experimentally studying both proximate and ultimate factors influencing animal personality (Groothuis & Carere 2005; Drent *et al*. 2003; van Oers *et al*. 2004, 2005; Dingemanse *et al*. 2004; Both *et al*. 2005). Selective breeding has clearly demonstrated that novelty-seeking behaviour in *P. major*, as quantified using early exploratory behaviour (EEB), has a significant genetic basis (Drent *et al*. 2003). Here, we describe evidence for an association between *P. major Drd4* gene polymorphisms and variation in novelty-seeking behaviour, as measured using EEB.

## 2. Material and methods

### (a) Birds studied

We investigated two categories of bird: unselected and EEB-selected. Unselected birds (*n*=91) were collected, as nestlings, from 17 nests in a natural population in The Netherlands and were hand-reared until independence (for details on housing and hand-rearing, see Drent *et al*. 2003). EEB-selected birds were from two lines bidirectionally selected, over four generations, for fast (*n*=29 individuals, 13 sibships) and slow (*n*=21 individuals, 8 sibships) early exploratory behaviour (EEB). Directly after independence, birds were scored for EEB based on two categories of behavioural test: (i) A novel environment exploration test conducted in a standard observation room. The time a bird took to visit a fourth tree within the room was converted into a scale of 0–10 (Drent *et al*. 2003). (ii) Two tests of the bird's reaction to two different novel objects placed in its home cage (Drent *et al*. 2003). The results of both novel object tests were converted into a scale of 0–5 (0, did not touch the perch on which the object was placed; 5, pecked the novel object). The final EEB score was the sum of all three tests giving an EEB score with a range of 0–20 (Drent *et al*. 2003).

### (b) Obtaining the P. major Drd4 orthologue cDNA sequence

A *P. major Drd4* cDNA sequence was obtained using 5′/3′-RACE (a detailed description of the methodology and the primers used is given in the electronic supplementary material accompanying this paper). Briefly, primers designed to anneal to evolutionarily conserved sequences, flanking the vertebrate *Drd4* third intracellular loop-coding region, were used to amplify this sequence from *P. major* genomic DNA. The resulting partial *Drd4* sequence was used to design primers to amplify a full-length *Drd4* cDNA sequence from *P. major* brain total RNA using a commercial 5′/3′-RACE kit (Roche Diagnostics, Germany).

### (c) Genotyping of the P. major Drd4 SNP830 polymorphism

A 152 bp region, flanking the C/T SNP830 of DQ006802, was amplified from *P. major* genomic DNA using a FAM-labelled forward primer (0.5 μM): 5′-6-FAM-AAGCTGAGAGGCTGCATCTATGG-3′ and the reverse primer (0.5 μM): 5′-ATCCCACTGTTCATCCCACACTC-3′ with reaction conditions: final volume 10.0 μl; 2 mM Mg^2+^; 94°C/4 min, one cycle; 94°C/30 s, 61°C/30 s, 72°C/30 s, 27 cycles; 72°C/1 min, one cycle, 4°C/hold catalysed using 1.25 U *Taq* DNA polymerase and its associated reaction buffer with (NH_4_)_2_SO_4_ (MBI Fermentas). The SNP830C/T resulted in the presence (5′-GC*C*GGC-3′) or absence (5′-GC*T*GGC-3′) of a *NaeI* restriction enzyme cleavage site and, consequently, a cleaved amplified polymorphic sequence assay was developed to discriminate between these two *Drd4* alleles, henceforth denoted *Drd4* SNP830C and SNP830T.

To provide a positive control for the *NaeI* cleavage reaction, a 172 bp sequence was amplified from a cloned *P. major Drd4* sequence carrying the SNP830C allele using forward primer: 5′-AACCTTGATGGAGAGAGAGCAGAC-3′ (unlabelled), VIC-labelled reverse primer: 5′-VIC-GTTGATCTTGGCCCGCTTGTG-3′ with reaction conditions as described earlier. PCR products generated from the DNA being genotyped (6-FAM-labelled, 152 bp) and the SNP830C positive control (VIC-labelled, 172 bp) were mixed in a ratio of 2 : 1 and digested using ‘Turbo’ *NaeI* (Promega). The resulting digestion products were diluted 1 : 400 in distilled water before 1.0 μl aliquots were resolved using an ABI PRISM 3100 Genetic Analyzer (Applied Biosystems, USA) with a molecular size standard (GeneScan-500 LIZ, Applied Biosystems). Sizes of the amplification products were determined using commercial software (Genotyper v. 1.1, Applied Biosystems). The 172 bp VIC-labelled positive-control PCR product was digested by *NaeI* to generate a single, VIC-labelled, fragment of 105 bp. The size and the number of 6-FAM-labelled fragments present after *NaeI* digestion were dependent upon the template DNA genotype: SNP830C/C homozygotes=one (117 bp) fragment; SNP830T/T homozygotes=one (152 bp) fragment and SNP830C/T heterozygotes=two (117 and 152 bp) fragments.

### (d) Detection of polymorphisms in P. major Drd4 genomic sequences

Using a combination of genomic walking and primers designed from the *P. major Drd4* cDNA sequence, *Drd4* sequences were amplified from genomic DNA isolated from two SNP830C/C (EEB scores of 6 and 0) and two SNP830T/T (EEB scores of 17 and 13) homozygous individuals. To correct for replication errors during amplification, each region was sequenced using products from multiple independent amplification reactions (figure 2 in electronic supplementary material). Sequences were aligned using BioEdit and examined for polymorphisms, defined as alternative sequences present in sequences obtained from two or more independent amplification reactions. Full details of the primers and amplification conditions used are given in the electronic supplementary material associated with this paper.

### (e) Genotyping of the P. major Drd4 polymorphic ID15 indel

Genotyping of the *Drd4* ID15 indel (±15 bp, coordinates 713–727 of DG006801) was performed using forward primer (0.5 μM): 5′-CCTCTGGAAGCAGAATTTGAGGA-3′ (corresponding to coordinates 514–536 of *DG006801*), and FAM-labelled reverse primer (0.5 μM): 5′-6FAM-GCCCCAAAGTTCCCTTACTCTT-3′ (coordinates 782–761 of *DG006801*). Reaction conditions: final volume of 10.0 μl; 2.0 mM Mg^2+^; 95°C/4 min; 95°C/30 s, 61°C/30 s, 72°C/30 s, 28 cycles; 72°C/30 s, 4°C/hold catalysed using 1.25 U *Taq* DNA polymerase and its associated reaction buffer with (NH_4_)_2_SO_4_ (MBI Fermentas). Amplification products (1.0 μl) were resolved using an ABI PRISM 3100 Genetic Analyzer (Applied Biosystems) along with a molecular size standard (GeneScan-500 LIZ, Applied Biosystems) and amplification product sizes calculated using the commercial software GeneScan v. 3.7 and Genotyper v. 3.6 (Applied Biosystems). The *Drd4* ID15+ and ID15- alleles generated amplification products of 269 and 254 bp, respectively.

### (f) Statistical analyses

To allow for the effects of pseudoreplication within sibships having the same mother, a randomization test was used to compare the genotype frequencies between the slow and fast EEB-selected lines. Complete sibships were randomly shuffled between the selected lines keeping the number of sibships per line constant and Fisher's exact test was used to test for differences in genotype frequencies between the lines. The observed *p*-value (without shuffling) was evaluated by the null distribution of *p*-values created by such 10 000 randomizations. The proportion of simulated *p*-values equal or lower than the observed *p*-value is reported as the empirical *p*-value. The same procedure was used to test for genotype differences between the unselected and selected lines. Among the unselected birds, there were multiple individuals from the same nests (same mother) and, therefore, the analyses of the unselected birds used a linear mixed-effects model approach with ‘nest of origin’ as the random factor. All three powerful association (effect inheritance) models with only one genotype factor (1 d.f.) were tested (Cordell & Clayton 2005): the two dominant/recessive models; and the trend model that tests for an allele dosage effect. Linkage disequilibrium was estimated from the unselected bird group by subsampling unrelated birds (one sib of each sibship) 10 000 times. Statistical calculations were performed using the software package R-2.3.1 (http://www.R-project.org). All tests are two-tailed with *α*=0.05.

### (g) Sequence analyses

DNA sequences were assembled using BioEdit (http://www.mbio.ncsu.edu/BioEdit/bioedit.html). Multiple protein sequence alignments were carried out using clustalw, as executed in BioEdit and Blast searches, and alignments were carried out using the online software available at http://www.ncbi.nlm.nih.gov/BLAST. Predicted protein transmembrane regions were identified using TMpred (http://www.ch.embnet.org/software/TMPRED_form.html). Possible transcription factor-binding sites were identified using Tfsearch (http://www.cbrc.jp/research/db/TFSEARCH.html).

## 3. Results and discussion

A cDNA sequence of the *P. major Drd4* orthologue was amplified, using 5′/3′-RACE, from brain total RNA. We concluded that the resulting cDNA sequence (1263 bp: 83 bp 5′UTR, 1095 bp ORF, 85 bp 3′UTR, GenBank acc. no. DQ006802) was the *P. major Drd4* orthologue on the basis of the following criteria: (i) a TBLASTN search of the GenBank (nr database) using the predicted 365 residue *P. major* DRD4 protein returned E-values of 4×*e*^−96^, 2×*e*^−91^ and 1×*e*^−153^ for alignments with the human (NM000797), mouse (NM007878) and chicken (XM420947) DRD4 protein sequences, respectively, (ii) a BLASTP search of the annotated predicted protein database associated with the *G. gallus* genomic sequence recovered a predicted dopamine D4 receptor with an E-value of 9×*e*^−156^, with the next closest homologies being with the predicted dopamine receptors D3 (1×*e*^−60^) and D2 (1×*e*^−52^), (iii) the predicted DRD4 protein was predicted to have the seven-transmembrane topology characteristic of G-protein-coupled receptors (data not shown), and (iv) the DRD4 protein showed 56% identity with the human DRD4 protein in contrast with identities of less than 40% for the four other recognized human dopamine receptors (electronic supplementary material, table 1) and phylogenetic trees constructed from these dopamine receptor sequences clustered the *P. major* predicted DRD4 sequence with the human DRD4 protein (details not shown).

Previous studies have reported that polymorphisms in the exon encoding the DRD4 receptor third intracellular loop are associated with personality variation among humans, monkeys and horses. Therefore, we examined the corresponding coding region in the *P. major Drd4* cDNA sequence (nucleotides 647–947 of DQ006802) for sequence polymorphisms. We directly sequenced PCR products amplified from genomic DNA from 27 individuals from two *P. major* lines selected over four generations for divergent levels of EEB (fast, i.e. having high EEB scores and slow, i.e. having low EEB scores; Drent *et al*. 2003). Only one single nucleotide polymorphism (SNP) was identified, corresponding to either C or T at position 830 of the *P. major Drd4* cDNA sequence (DQ006802). Frequencies of the three *Drd4* SNP830 genotypes differed significantly between birds of the slow (mean EEB score=0.81±1.40 s.d., *n*=21) and fast EEB (mean EEB score=14.55±3.31 s.d., *n*=29) lines, with the slow EEB line having a lower SNP830T allele frequency than the fast line (figure 1*a*). Furthermore, the SNP830 genotype frequencies in the slow EEB line, but not in the fast EEB line, differed significantly from genotype frequencies in unselected birds taken from a Dutch natural *P. major* population, with the SNP830T/T and SNP830C/T genotypes being scarcer in the slow EEB line than among the unselected birds (table 1). These results are consistent with the EEB phenotype-based selection regime having selected against the SNP830T allele in the slow EEB selection line. However, the relative scarcity of the SNP830T allele in the slow EEB selection line could also be a consequence of simple random genetic effects, such as founder effects and genetic drift, within the small populations forming the EEB-selected lines (Drent *et al*. 2003). To address this problem, we investigated the association between SNP830 genotypes and EEB score among unselected birds taken from a natural population.

Among the unselected birds, differences in EEB scores were associated with differences in *Drd4* SNP830 genotype: the mean EEB score of SNP830C/C homozygotes was significantly lower than the means of both SNP830C/T heterozygotes and SNP830T/T homozygotes (figure 2). This finding suggests a dominance effect of the SNP830T allele, which is consistent with the SNP830 genotype frequencies found in the EEB-selected lines. Thus, selecting for the slow EEB phenotype may have resulted in selection against both SNP830C/T heterozygotes and SNP830T/T homozygotes, leading to the observed lower frequency of the SNP830T allele in the slow EEB line (figure 1*a*).

These results indicate a statistically significant association between the *P. major Drd4* SNP830 genotype and EEB scores. Such correlational findings, however, do not prove any functional link between the analysed locus and phenotype. Indeed, the SNP830 polymorphism, located at the third base of codon 249, is synonymous, with both alleles encoding Ala_249_. Examination of an avian (*G. gallus*) codon usage table (http://www.kazusa.or.jp/codon/cgi-bin/showcodon.cgi?species=Gallus+gallus+[gbvrt]) indicated that the alternative Ala_249_ codons would be translated with similar efficiency. Although a phenotypic effect through the protein structure of the *Drd4* SNP830 polymorphism can be excluded, it is noteworthy that synonymous polymorphisms have been shown to affect transcription, splicing, mRNA stability and translation, in general (Chamary *et al*. 2006; Goymer 2007), and in dopamine receptors, in particular (Duan *et al*. 2003).

The association between SNP830 genotype and EEB phenotype might be explained by linkage disequilibrium between SNP830 variants and one or more functionally significant polymorphism(s) which could possibly be located within the *Drd4* gene itself. In a search for such additional polymorphisms, *P. major Drd4* genomic sequences were obtained from a minimum of two SNP830C/C and two SNP830T/T homozygous genomes with a representative (SNP830C) sequence deposited on GenBank (10897 bp, acc. no. DQ006801; figure 2 in electronic supplementary material). Alignment of the *Drd4* genomic (DQ006801) and cDNA (DQ006802) sequences allowed definitive identification of exon/intron boundaries, while a possible transcription initiation region was tentatively assigned as being adjacent to the 5′ end of the 5′-RACE products (i.e. more than 1763 of DQ006801) (figure 3 and figure 2 in electronic supplementary material).

The *P. major Drd4* gene, like its human orthologue, is clearly highly polymorphic (figure 3; Cravchik & Goldman 2000). By comparing the *Drd4* SNP830C and SNP830T genomic sequences, we identified 73 polymorphisms: 66 SNPs and 7 indels (figure 3), which were grouped for declining degrees of association with the SNP830 polymorphisms: category-1, positions where the alternative sequences were associated exclusively with either the SNP830C or SNP830T allele; category-2, positions where either the SNP830C or the SNP830T sequences, but not both, were heterogeneous; and category-3, positions where both the SNP830C and the SNP830T allelic sequences were heterogeneous for the polymorphism. No additional polymorphisms were found within the *Drd4*-coding region and a single C/T SNP in the 5′UTR showed no evidence of an association with either the SNP830C or SNP830T polymorphism (i.e. category-3; figure 3). All 21 category-1 polymorphisms were SNPs located within introns and, on the basis of their nature and position, functional significance could not be attributed to these variants. Among the 47 polymorphisms in category-2, we identified an indel of possible functional significance. A 15 bp indel, located 1036 bp from the tentatively assigned transcription initiation site (coordinates 713–727 of DQ006801; ID15, figure 3), was predicted by transcription factor-binding site identification software (Tfsearch) to potentially result in the presence or absence of two putative transcription factor-binding sites. As polymorphisms in the *Drd4* promoter region had previously been associated with variation in human personality traits (Kluger *et al*. 2002; Schinka *et al*. 2002; Reif & Lesch 2003; van Gestel & Van Broeckhoven 2003; Savitz & Ramesar 2004; Ebstein 2006), we decided to determine if there was any evidence for an association between the *P. major Drd4* ID15 polymorphism and EEB score.

*Drd4* ID15 genotypes of both EEB-selected and unselected birds were determined on the basis of the size of amplification products generated using primers flanking the ID15 polymorphism. The slow and fast EEB lines did not differ significantly in the frequencies of the three ID15 genotypes (figure 1*b*). However, the slow EEB line did differ significantly in ID15 genotype frequencies when compared with the unselected birds (table 1). It is not surprising that the slow EEB line differed significantly from the unselected birds in both the SNP830 and ID15 genotype frequencies, because the two polymorphic sites are separated by only 9359 bp and selection was only over four generations. Therefore, any allelic associations between polymorphisms (i.e. haplotype combinations) in the selection line founder birds are likely to remain among their descendents.

Among the unselected birds, no association was detected between EEB score and *Drd4* ID15 genotype. However, a statistically significant interaction was found between the ID15 and SNP830 genotypes (mixed-effects model: *p*=0.016). Thus, the association between the absence of a SNP830T allele and lower EEB scores described earlier (figure 2) was predominantly found in those unselected birds that carried at least one copy of the ID15 (i.e. short) allele (figure 3 in electronic supplementary material). We speculate that genotypes combining *Drd4* haplotypes with particular ID15 and SNP830-associated polymorphisms may account for most of the association between *Drd4* genotype and EEB score observed in this study. Using the genotype data of 17 unselected birds, each randomly selected from within a different sibling group, yielded a median *r*^2^ value (a measure of linkage disequilibrium, LD) of 0.029 indicating no significant LD between the SNP830 and ID15 polymorphisms among the unselected birds. In conclusion, although the observed interaction was statistically significant, its biological importance remains to be shown.

## 4. Conclusion

We have obtained evidence, from a free-living bird species, supporting the hypothesis that *Drd4* gene polymorphisms are associated with variation in the level of exploratory/novelty-seeking behaviour in vertebrates. The two sequence polymorphisms for which we found an association with EEB are located in regions of the *Drd4* gene, the promoter (ID15) and third intracellular loop-coding region (SNP830), that harbour personality-associated variants in humans, monkeys and horses (Kluger *et al*. 2002; Schinka *et al*. 2002; Reif & Lesch 2003; van Gestel & Van Broeckhoven 2003; Savitz & Ramesar 2004; Momozawa *et al*. 2005; Ebstein 2006; Bailey *et al*. 2007). Thus, differing types of sequence polymorphisms (variable number tandem repeats, SNPs and indels), located in similar *Drd4* gene regions (e.g. third intracellular loop-coding region, promoter region), may be associated with variation in similar personality traits among taxonomically diverse vertebrates. Indeed, the results reported here suggest that a general association between *Drd4* gene polymorphisms and animal personality variation predates the divergence of the avian and mammalian lineages. Although the association between the SNP830 and EEB was found in both the selected lines and in the wild population of great tits, we cannot entirely rule out that the observed signal is due to cryptic population stratification. This could be further investigated by analysing other non-candidate, unlinked markers (preferably SNPs), that have to be developed first.

This work opens up the exciting possibility of following changes in *P. major* *Drd4* allele frequencies within free-living bird populations, where natural selection is known to act differentially on different personality types. Such studies would help to gain insights into the selective pressures that maintain animal personality trait variation.

## Figures and Tables

**Figure 1:**
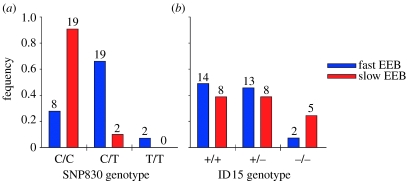
Proportions of *P. major* individuals of differing *Drd4* genotypes in two lines selected for divergent levels of early exploratory behaviour (EEB). The numbers above the bars indicate the total number of individuals, including groups of full sibs. Note that the statistical analysis controls for the presence of sibships (see text for details). (*a*) *Drd4* SNP830 genotype frequencies differed significantly between the fast and slow EEB-selected lines (randomization test, empirical *p*=0.004). (*b*) *Drd4* ID15 genotype frequencies did not differ significantly between the EEB-selected lines (randomization test, empirical *p*=0.49).

**Figure 2:**
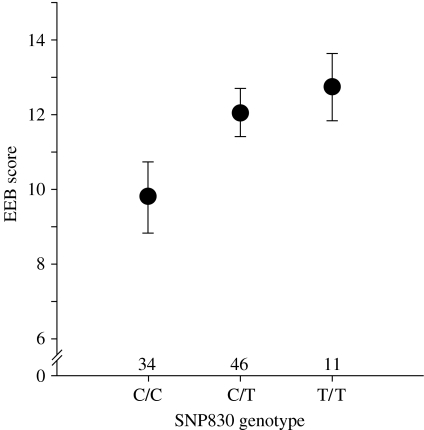
EEB scores of unselected birds genotyped for the *Drd4* SNP830 polymorphism. Data shown are mean EEB values ±s.e.m.; sample sizes are indicated above the *x*-axis and refer to the total number of individuals including offspring from the same brood. Note that the statistical analysis (mixed-effects models) controls for the presence of these siblings (see text for details). The most prominent difference in EEB scores is between the SNP830C/C birds and the other two genotypes, indicating a partially dominant effect of the SNP830T allele. Overall, EEB score differed significantly among SNP830 genotypes in both the effect trend model (*p*=0.038) and the dominant effect model with SNP830T as the dominant allele (*p*=0.030).

**Figure 3:**
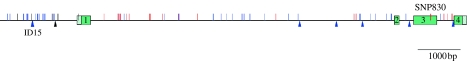
Schematic of the *P. major Drd4* gene structure. Exons are shown as green boxes (coding regions full colour, untranslated regions striped). A total of 73 polymorphisms were identified (66 SNPs: vertical lines; 7 indels: triangles). The polymorphisms were categorized according to decreasing association with SNP830 alleles (see text): category-1, red; category-2, blue; category-3, black.

**Table 1:** Frequencies of *P. major Drd4* SNP830 and ID15 genotypes among unselected birds and among the two EEB-selected lines. (Figures shown are proportions with number of individuals bracketed. *p*-values are based on randomization tests for differences between the unselected and EEB-selected lines.)

	SNP830 genotype			*p*
group	C/C	C/T	T/T	
unselected	0.37 (34)	0.51 (46)	0.12(11)	
fast EEB	0.28 (8)	0.66 (19)	0.07 (2)	0.69
slow EEB	0.90 (19)	0.10 (2)	0.00 (0)	0.017
	ID15 genotype			*p*
group	+/+	+/−	−/−	
unselected	0.66 (59)	0.33 (29)	0.01 (1)	
fast EEB	0.48 (14)	0.45 (13)	0.07(2)	0.15
slow EEB	0.38 (8)	0.38 (8)	0.24 (5)	0.024

## Supplementary Material

Electronic supplementary material 1—Materials and methods Contains additional details on the methodology

Electronic supplementary material 2—interaction between SNP830 and ID15 polymorphisms Additional figure
