# Supplementary material for: Drd4 gene polymorphisms are associated with personality variation in a passerine bird
Source: Proc Biol Sci. 2007 May 1;274(1619):1685–91. doi: 10.1098/rspb.2007.0337 (PMC1914334; doi:10.1098/rspb.2007.0337)
Supplement: Supplementary file 1 [file rspb20070337s02.doc]

Electronic Supplementary Material (ESM)

*Drd4* gene polymorphisms are associated with personality variation in a passerine bird. Fidler *et al.*

ESM 1 - Materials and Methods

**Amplification of *P. major Drd4* orthologue cDNA sequences.** The avian *Drd4* gene structure was assumed to be similar to that of previously reported mammalian *Drd4* genes (O’Malley *et al.* 1992; Fishburn *et al.* 1995). In particular it was assumed that the third intracellular loop of the avian DRD4 protein is encoded by a single exon. TBLASTN searches of the GenBank EST database, using three mammalian DRD4 protein sequences (mouse: acc. no. **U19880**, rat: acc. no. **M84009**, human: acc. no. **L12398**), identified two chicken ESTs (GenBank acc. no.s: **BU460774** and **BU373433**) encoding putative avian *Drd4* orthologues. These EST sequences were used to design a primer pair (forward: 5’ – TGCTCCTTCTTCATCCCTTGCCC –3’, reverse: 5’– GGCAGTACCCGCATGGCCTT –3’) predicted to flank the *Drd4* third intracellular loop coding region and to generate an amplification product of approximately 0.3 kb. *Taq* DNA polymerase (Roche Diagnostics, Germany) catalyzed PCR was carried out, using *P. major* genomic DNA as template, with reaction conditions: 1.5 mM Mg^2+^; 94^o^C / 2 minutes; 94^o^C / 30 seconds, 57^o^C / 30 seconds, 72^o^C / 60 seconds; 10 cycles; 94^o^C / 30 seconds, 60^o^C / 30 seconds, 72^o^C / 60 seconds incrementing 5 seconds /cycle, 30 cycles; 72^o^C / 7 minutes; 4^o^C / hold. An amplification product of 0.3 kb was cloned into T-tailed cloning vector (pGEM-Teasy, Promega, Madison, U.S.A.) and sequenced by an external contractor (MWG Biotech, Ebersberg, Germany). Sequence identity was investigated using both BLASTN and TBLASTN searches of GenBank. To obtain a corresponding full-length cDNA sequence total RNA was purified from *P. major* brain tissue using Trizol^TM^ reagent (Invitrogen, Carlsbad, U.S.A.). For 3’RACE first strand cDNA was generated using the reverse primer: 5’- GAGGACTCGAGCTCAAGCCCAGTGAGCAGAGTGACGT_18_ -3’ and AMV reverse transcriptase (Roche Diagnostics). A first round of PCR amplification was carried out using a *Drd4* gene specific forward primer (5’- GGTGCTGTACTGTGGCATGTTCC –3’) designed from the 0.3 kb *Drd4* fragment described above, and the reverse primer: 5’- GAGGACTCGAGCTCAAGCCCA -3’. PCR conditions (Expand Long Template PCR System, Roche Diagnostics), 1.75 mM Mg^2+^; 94°C, 4 minutes; 94°C / 30 seconds, 63°C / 30 seconds, 68°C / 3 minutes, 10 cycles; 94°C / 30 seconds, 62°C / 30 seconds, 68°C / 3 minutes incrementing 5 seconds/cycle, 25 cycles; 68°C / 4 minutes; 4°C / hold. The PCR mixture was diluted 1:20 in water and 1.0 µl used as template for a nested PCR with a second *Drd4* gene specific forward primer (5’- GCCAACAGGAAGCTGTATCACC –3’) and a nested reverse primer (5’ - AGCCCAGTGAGCAGAGTGACG -3’). PCR conditions were as for the first round except with annealing temperatures of 62°C for the first 10 cycles and 61°C for the following 25 cycles. An amplification product of approximately 0.6 kb was cloned and sequenced. Obtaining the 5’ region sequence of the *Drd4* mRNA required two 5’-RACE steps. First 5'-RACE procedure: first strand cDNA was generated from great tit brain total RNA using a *Drd4* gene specific reverse primer (5'- GTTGATCTTGGCCCGCTTGT -3') and using a commercial 5’/3’ RACE kit (Roche Diagnostics). The resulting cDNA was purified, and after adding a poly-dA tail, used as template for a PCR with a second *Drd4* gene specific reverse primer (5'- TCCCACTGTTCATCCCACACTC -3') and the poly-dT-anchored forward primer supplied with the 5’/3’RACE kit. PCR was performed using the GC-RICH PCR System (Roche Diagnostics) with 0.5M CG-RICH resolution solution and the cycling conditions: 95°C / 4 minutes; 95°C / 30 seconds, 63°C / 30 seconds, 72°C / 2 minutes incrementing 5 seconds/cycle, 10 cycles; 95°C / 30 seconds, 62°C / 30 seconds, 72°C / 2 minutes incrementing 5 seconds/cycle, 25 cycles; 72°C/ 4 minutes; 4°C / hold. The resulting PCR product was purified (High Pure PCR Purification kit, Roche Diagnostics), diluted 1:20 and 1.0 µl of the dilution used as template in a nested PCR with a nested *Drd4* gene specific reverse primer (5’- TCCCACTGTTCATCCCACACTC -3’) and the nested forward anchor primer supplied with the RACE kit. PCR conditions were as for the first round of 5’-RACE except using annealing temperatures of 62°C for the first 10 cycles and 61°C for the following 25 cycles. An amplified product of 0.7 kb was cloned and sequenced and determined to be a 5’ extension of the known mRNA sequence. However, as this 0.7 kb sequence did not include a possible start codon, a second 5’RACE was performed to obtain further 5’ *Drd4* cDNA sequences. The procedure used was essentially the same as for the first 5’RACE except in having an additional round of nested PCR and in sequentially using the following four gene specific reverse primers: (a) for cDNA synthesis: 5’- GGGGTGATACAGCTTCCTGTTG -3’, (b) for the first PCR: 5'- ATCAGGGCATCGCACAGCAC -3', (c) for the first nested PCR: 5'- TGCAGACGCTCAGACAGACGA -3' and (d) for the second nested PCR: 5'- CGATGAGGAGGATGAGGAGGA –3'. All three PCRs used the same cycling conditions: 95°C / 4 minutes, 1 cycle; 95°C / 30 seconds, 61°C / 30 seconds, 72°C / 2 minutes incrementing 5 seconds/cycle, 10 cycles; 95°C / 30 seconds, 59°C / 30 seconds, 72°C / 2 minutes incrementing 5 seconds/cycle, 25 cycles; 72°C / 4 minutes; 4°C / hold. An amplification product of 0.2 kb, generated by the second nested PCR, was sequenced and shown to encode sequences extending 5’ beyond a predicted start codon.

**Detection of polymorphisms in *P. major Drd4* genomic sequences.** Allelic (i.e. SNP830C-associated and SNP830T-associated) *Drd4* sequences were obtained by PCR amplification from both *Drd4* SNP830C/C and SNP830T/T homozygous genomes previously genotyped using the NaeI-based cleaved amplified polymorphic sequence (CAPS) procedure. Due to the length of the *P. major Drd4* gene, its genomic sequence was obtained in three stages, using a combination of genomic walking and PCR with primers annealing to exon sequences obtained from the *Drd4* cDNA sequence: (i) exon 2 – exon 4 sequence, (ii) intron1 sequence and (iii) 5’-region / exon1 (ESM Figure 1).

(i) Sequences between the middle of exon 2 and the 3’ end of exon 4 were amplified using primers designed from the *Drd4* cDNA sequence; forward primer: 5’- GAGGAGTGTGGTCCCTCAGC -3’, reverse primer: 5’- CGCAGAAATAGACCTTTAATGAACTATAC -3’, using the GC-Rich PCR System (Roche Diagnostics) and reaction conditions: 95°C / 4 minutes; 95°C / 30 seconds, 59°C / 30 seconds, 68°C / 2.5 minutes, 10 cycles; 95°C / 30 seconds, 57°C / 30 seconds, 68°C / 2.5 minutes, 25 cycles; 68°C / 2.5 minutes, 4°C / hold. The 1.6 kb region amplified is indicated in ESM Figure 1.

(ii) The length of the *Drd4* intron1 (i.e. > 7 kb) complicated PCR amplification which was performed in four steps.

(ii, a) A 5’ genomic walk, from exon2, was performed using the Universal Genome Walker kit (Clontech, Palo Alto, U.S.A.) following the manufacturers’ instructions. The first round of PCR used the gene specific reverse primer: 5'- ATCCACGCTGATAGCACACAGGTTGAAGAT -3', in combination with primer-1 supplied with the kit and reaction conditions: 94°C / 2 seconds, 72°C / 4.5 minutes, 7 cycles; 94°C / 2 seconds, 67°C / 4.5 minutes, 32 cycles; 67°C / 4 minutes; 4°C / hold. Amplification products were diluted 1:49 and used as templates for nested PCRs using a gene specific reverse primer: 5'- CATGGTCATCAGGGCATCGCACAGCA -3', a nested primer-2 supplied with the kit and reaction conditions: 94°C / 2 seconds, 72°C / 4.5 minutes, 5 cycles; 94°C / 2 seconds, 67°C / 4.5 minutes, 20 cycles; 67°C / 4 minutes; 4°C / hold. A 2.7 kb amplification product was sequenced and shown to include 31 bp of *Drd4* exon2 sequence at its 3’ end (ESM Figure 1).

(ii, b) A reverse primer (5'- TCAGCCCATTCTGGTATCCTTATTCCTAAGCA -3') was designed from the 5’ end of the 2.7 kb 5’-walk product and used in combination with a forward primer designed from the *Drd4* cDNA exon1 sequence (5'- TCGGCATCCTCCTCATCCTC -3') using the GC-Rich PCR System (Roche Diagnostics) and reaction conditions: 95°C / 4 minutes; 95°C / 30 seconds, 62°C / 30 seconds, 68°C / 3.5 minutes, 10 cycles; 95°C / 30 seconds, 60°C / 30 seconds, 68°C / 3.5 minutes, 22 cycles; 68°C / 3.5 minutes; 4°C / hold. An amplification product of 4.7 kb was obtained (ESM Figure 1).

(ii, c) A forward primer was designed from the 2.7 kb sequence (5'- AAGTTCTCTCCTAGCACCTTTC -3') and paired with an exon2 reverse primer (5'- ATCAGGGCATCGCACAGCAC -3') to amplify 2.1 kb sequences with reaction conditions: 95°C / 4 minutes; 95°C / 30 seconds, 58°C / 30 seconds, 68°C / 2 minutes, 10 cycles; 95°C / 30 seconds, 56°C / 30 seconds, 68°C / 2 minutes, 20 cycles; 68°C / 2 minutes; 4°C / hold (ESM Figure 1).

(ii, d) A gap between the sequences generated in the second (ii, b) and third step (ii, c) was bridged using forward primer: 5'-TCGGTTCTGTCCTGGCTCAT -3', reverse primer: 5'-GCAGCACCTTTGGATCATGTG -3'; reaction conditions: 95°C 3 minutes; 95°C / 30 seconds, 60°C / 30 seconds, 72°C / 30 seconds, 5 cycles; 95°C / 30 seconds, 58°C / 30 seconds, 72°C / 30 seconds, 25 cycles; 72°C / 30 seconds, 4°C / hold, producing am amplification product of 0.6 kb (ESM Figure 1).

(iii) 5’-region / exon1 sequences were obtained using the Universal Genome Walker kit (Clontech) and following the manufacturers’ instructions. The two gene specific primers used were: first PCR: 5'- GGCTTTGACCCTCGGCACTTGGTCTCTT -3', second (nested) PCR 5'- GGCGAGGCTGACGATGAAGTAGTTGGT -3'. Reaction conditions for the first PCR were: 95°C / 2 minutes, 95°C / 20 seconds, 72°C / 4 minutes, 7 cycles; 95°C / 20 seconds, 67°C / 4 minutes, 32 cycles; 67°C / 4 minutes; 4°C / hold; conditions for the second (nested) PCR: 95°C / 2 minutes, 95°C / 20 seconds, 72°C / 4 minutes, 5 cycles; 95°C / 20 seconds, 67°C / 4 minutes, 20 cycles; 67°C / 4 minutes; 4°C / hold. A 2.1 kb amplification product (ESM Figure 1) had 233 bp of 3’ sequence which aligned with the *Drd4* cDNA sequence. The 2.1 kb was used to design a forward primer: 5’- GGGCCCCCTTTTACTACTTTGAGCTGATTT -3’ which was used in combination with the reverse primer: 5’- GGCTTTGACCCTCGGCACTTGGTCTCTT -3’ and reactions conditions: GC-Rich PCR system (Roche Diagnostics), 95°C / 4 minutes; 95°C / 30 seconds, 67°C / 30 seconds, 72°C / 2 minutes, 10 cycles; 95°C / 30 seconds, 65°C / 30 seconds, 72°C / 2 minutes, 20 cycles; 72°C / 2 minutes; 4°C / hold.

**ESM Figure 1.** Schematic summary of sequences generated in the search for *P. major Drd4* polymorphisms. *Drd4* genomic sequences were amplified using the PCR. Regions amplified once following 5’ genomic walks are indicated by dotted black lines while sequences amplified and sequenced multiple times are indicated by solid blue lines. Bird identification numbers and SNP830 genotypes are shown adjacent each amplified region: SNP830C/C = green text, SNP830T/T = red text. The number of independent amplification reactions from each genome is indicated in brackets. Exons are indicated by green boxes, introns by thin black lines. ATG = predicted translation start codon, TGA = predicted translation stop codon. Details of the amplification procedures are given above. *Abbreviations*: C/C = SNP830C/C; T/T = SNP830T/T; ex = exon; in = intron.

**ESM Figure 2.** Alignment of the *P. major* predicted DRD4 protein sequence with established avian and mammalian DRD4 sequences. Sequences were aligned using ClustalW with additional manual alignments. Positions of identity between all the aligned sequences are indicated by asterisks and seven predicted transmembrane regions are indicated by blue bars. GenBank accession numbers and their percent identity / similarity with *P. major* DRD4 (**DQ006802**) calculated using BLAST2 / BLASTP (filter feature disabled, BLOSUM62 matrix) are as follows. *Gallus gallus* isolate 9 (*Gg*i9) (**AB125363**): 63 amino acids, 93% / 96%; *Gallus gallus* (*Gg*) (**XM_420947**) C-terminal 326 amino acids predicted from the *G. gallus* genome (build 1.1): 87% / 90%; *Mustela putorius (Mp)* (**AY394848**): 60% / 71%; *Mus musculus* (*Mm*) (**NM_007878**): 55% / 66%; *Rattus norwegicus (Rn)* (**U03551**): 55% / 66%; *Homo sapiens (Hs)* (**NM_000797**): 56% / 65%. Note that the N-terminal portion of the *G. gallus* DRD4 sequence, as predicted by automated computational analysis (GNOMON) of the draft chicken genome sequence (build 1.1), (**XM_420947**) was judged to be incorrect and, consequently, only 326 amino acids of the predicted protein was used in this alignment. *Gg*i9 (**AB125363**) represents 63 residues of a published predicted *G. gallus* N-terminal DRD4 sequence.

#####	TM1	TM2	TM3

```
     ….........................................................................................................................................
Pm   MGNGTAG~~~~~~~~~~PPPAGAG~~~~~~~~~HSIAALVLGILLILLIVGGNGLVCLSVCTERALKTTTNYFIVSLAVADLLLALLVLPLYVYSEFQGGVWSLSTVLCDALMTMDVMLCTASIFNLCAISVDRFIAVQI 121
Ggi9 ~~~~~~~~~~~~~PPPPPPPPPAG~~~~~~~~~HNIAALVLGIVLILLIVGGNGLVCLSVCTERALKTTTNYFIVSLAVADLLLA~~~~~~~~~~~~~~~~~~~~~~~~~~~~~~~~~~~~~~~~~~~~~~~~~~~~~~~ 63
Gg   ~~~~~~~~~~~~~~~~~~~~~~~~~~~~~~~~~~~~~~~~~~~~~~~~~~~~~~~~~~~VCTERALKTTTNYFIVSLAVADLLLALLVLPLYVYSEFQGGVWSLSTVLCDALMTMDVMLCTASIFNLCAISVDRFIAVSV 81
Mp   MGNRSAADADGLLAGRGP~GTGGGAGSPG~~~~~AAAALVGGVLLIGAVLAGNALVCVSVAAERALQTPTNYFIVSLAAADLLLALLVLPLFVYSEVQGGVWQFSPGLCDALMAMDVMLCTASIFNLCAISADRFVAVAV 134
Mm   MGNSSATEDGGLLAGRGPESLGTGAGLGG~~~~AGAAALVGGVLLIGLVLAGNSLVCVSVASERTLQTPTNYFIVSLAAADLLLAVLVLPLFVYSEVQGGVWLLSPRLCDTLMAMDVMLCTASIFNLCAISVDRFVAVTV 136
Rn   MGNSSATGDGGLLAGRGPESLGTGTGLGG~~~~AGAAALVGGVLLIGMVLAGNSLVCVSVASERILQTPTNYFIVSLAAADLLLAVLVLPLFVYSEVQGGVWLLSPRLCDTLMAMDVMLCTASIFNLCAISVDRFVAVTV 136
Hs   MGNRSTADADGLLAGRGP~AAGASAGASAGLAGQGAAALVGGVLLIGAVLAGNSLVCVSVATERALQTPTNSFIVSLAAADLLLALLVLPLFVYSEVQGGAWLLSPRLCDALMAMDVMLCTASIFNLCAISVDRFVAVAV 139
     ***              *                  **** *  **     ** *** **  ** * * ** ****** ****** ***** **** *** *  *  *** ** ***************** *** **


                            TM4                                   TM5
     ….........................................................................................................................................
Pm   PLNYNRRQIDLRQLILISTTWIFAFAVASPVIFGLNNVPNRDPSLCQLEDDNYIVYSSICSFFIPCPVMLVLYCGMFQGLKRWEEARKAKLRGCIYGANRKLYHPP~~~~~TLMEREQTRLGLLDCSSPYARAG~~LPGE 254
Gg   PLNYNRRQIDLRQLILISTTWIFAFAVASPVIFGLNNVPNRDPSLCQLEDDNYIVYSSICSFFIPCPVMLVLYCAMFQGLKRWEEARKAKLRGGIYGGNRMLYHPS~~~~~PFIERERVGMEPEEYH~PYAHPEHPLSGD 215
Mp   PLSYNRQSGGGRQLLLIGATWLLSAAVAAPVLCGLNDARGRDPAVCRLEDRDYVVYSSVCSFFLPCPVMLLLYWATFRGLRRWEAARRTKLHGRRPRRPSGPGPPP~~~~~PEAVETPEAPEAIP~~~~~~~~~~TPDAT 259
Mm   PLRYNQQG~~QCQLLLIAATWLLSAAVASPVVCGLNDVPGRDPAVCCLENRDYVVYSSVCSFFLPCPLMLLLYWATFRGLRRWEAARHTKLHSRAPRRPSGPGPPV~~~~~SDPTQGPFFPDCPPPLPSLRTS~~PSDSS 267
Rn   PLRYNQQG~~QCQLLLIAATWLLSAAVAAPVVCGLNDVPGRDPTVCCLEDRDYVVYSSICSFFLPCPLMLLLYWATFRGLRRWEAARHTKLHSRAPRRPSGPGPPV~~~~~SDPTQGPLFSDCPPPSPSLRTS~~PTVSS 267
Hs   PLRYNRQGGSRRQLLLIGATWLLSAAVAAPVLCGLNDVRGRDPAVCRLEDRDYVVYSSVCSFFLPCPLMLLLYWATFRGLQRWEVARRAKLHGRAPRRPSGPGPPSPTPPAPRLPQDPCGPDCAPPAPGLPRGPCGPDCA 279
     ** **       ** **  **    *** **  ***    *** *  **   * **** **** *** ** **   * ** *** **  **             *


                                                                                   TM6                            TM7
     ….........................................................................................................................................
Pm   CGMNSGIQTVSYPHLRYPHPG~~~~~~~~~~~~~~~~~~~~~~~~~~~~~HGHKRAKINGRERKAMRVLPVVVGAFLFCWTPFFVVHITRALCKSCSIPPQVTSTVTWLGYVNSALNPIIYTVFNAEFRNFFRKVLHVFC 365
Gg   YVMSNGLQTVSYPHLKYPHPA~~~~~~~~~~~~~~~~~~~~~~~~~~~~~HGQKRAKINGRERKAMRVLPVVVGAFLFCWTPFFVVHITRALCKSCTIPTQVTSIVTWLGYVNSAVNPIIYTVFNAEFRNFFRKVLHLFC 326
Mp   LAEPALPAS~~~~~~~~~~~~~~~~~~~~~~~~~~~~~~~~~~~~~~~~~~EERRAKITGRERKAMRVLPVVVGAFLVCWTPFFVVHITGALCPACAVPPRLVSAVTWLGYVNSALNPLIYTVFNAEFRAVFRKALRLCC 357
Mm   RPESELSQRPCSPGCLLADAALPQPP~~~~~~~~~~~~~~~~~~~~EPSSRRRRGAKITGRERKAMRVLPVVVGAFLVCWTPFFVVHITRALCPACFVSPRLVSAVTWLGYVNSALNPIIYTIFNAEFRSVFRKTLRLRC 387
Rn   RPESDLSQSPCSPGCLLPDAALAQPP~~~~~~~~~~~~~~~~~~~~APSSRRKRGAKITGRERKAMRVLPVVVGAFLMCWTPFFVVHITRALCPACFVSPRLVSAVTWLGYVNSALNPIIYTIFNAEFRSVFRKTLRLRC 387
Hs   PAAPSLPQDPCGPDCAPPAPGLPPDPCGSNCAPPDAVRAAALPPQTPPQTRRRRRAKITGRERKAMRVLPVVVGAFLLCWTPFFVVHITQALCPACSVPPRLVSAVTWLGYVNSALNPVIYTVFNAEFRNVFRKALRACC 419
                                                            *** ****************** *********** ***  *       * ********** ** *** ******  *** *   *
```

| ESM Table 1 Quantitative comparisons of the predicted *P. major* DRD4 protein sequence with the five known human dopamine receptor protein sequences. | | | |
| --- | --- | --- | --- |
| Human DR protein | Comparison with *P. major* | | |
|  | % Identity | % Similarity | % Gaps |
| D1 | 31 | 45 | 17 |
| D2 | 38 | 53 | 18 |
| D3 | 39 | 57 | 8 |
| D4 | 56 | 65 | 12 |
| D5 | 27 | 44 | 22 |

Using BLAST2 / BLASTP (BLOSUM62 matrix, filter function disabled) the predicted *P. major* DRD4 sequence was aligned with each of five human dopamine receptor proteins and percent identities, similarities and gaps calculated. Human dopamine receptor GenBank accession numbers: DRD1: **NM_000794**, DRD2: **NM_000795**, DRD3: **NM_000796**, DRD4: **NM_000797**, DRD5: **NM_000798**.

**ESM References**

Fishburn, C. S., Carmon, S. & Fuchs, S. 1995 Molecular cloning and characterisation of the gene encoding the murine D4 dopamine receptor. *FEBS Lett.* **361**, 215-219.

O’Malley, K. L., Harmon, S., Tang, L. & Todd, R. D. 1992 The rat dopamine D4 receptor: sequence, gene structure, and demonstration of expression in the cardiovascular system. *New Biol.* **4**, 137-146.
