# Supplementary material for: Drd4 gene polymorphisms are associated with personality variation in a passerine bird
Source: Proc Biol Sci. 2007 May 1;274(1619):1685–91. doi: 10.1098/rspb.2007.0337 (PMC1914334; doi:10.1098/rspb.2007.0337)
Supplement: Supplementary file 2 [file rspb20070337s03.doc]

Electronic Supplementary Material (ESM)

*Drd4* gene polymorphisms are associated with personality variation in a passerine bird. Fidler *et al.*

ESM 2 - Interaction between SNP830 and ID15 polymorphisms

**Figure.** EEB scores of unselected birds genotyped for both the *Drd4* SNP830 and ID15 polymorphisms. Data shown are mean EEB values ± s.e.m. Groups represent individuals with or without the SNP830T allele and with or without the ID15- allele. The interaction between the SNP830 and the ID15 polymorphisms was significant in a mixed-effects model (P = 0.016).
